# (*E*)-2,4-Dihydroxy­benzaldehyde 4-ethyl­thio­semicarbazone–4,4′-bipyridine–water (4/7/2)

**DOI:** 10.1107/S1600536809030852

**Published:** 2009-08-12

**Authors:** Seik Weng Ng

**Affiliations:** a Department of Chemistry, University of Malaya, 50603 Kuala Lumpur, Malaysia

## Abstract

The asymmetric unit of the title compound, 7C_10_H_8_N_2_·4C_10_H_13_N_3_O_2_S·2H_2_O, contains two independent 2,4-hydroxy­benzaldehyde 4-ethyl­thio­semicarbazone mol­ecules, three and a half 4,4′-bipyridine mol­ecules and one water mol­ecule. Two of the 4,4′-bipyridine mol­ecules lie on general positions and the other three on centers of inversion. The two 4,4′-bipyridine mol­ecules on general positions and one of the three on special positions are disordered over two positions each with an occupancy of 0.50. The –NH—C(=S)—NH—NC fragment is close to planar in the two 2,4-hydroxy­benzaldehyde 4-ethyl­thio­semicarbazone mol­ecules (r.m.s. deviations = 0.04 and 0.05 Å). In the crystal, the Schiff base, *N*-heterocycle and water mol­ecules engage in O—H⋯O, O—H⋯N and N—H⋯O hydrogen-bonding inter­actions, generating a layer structure.

## Related literature

4,4′-Bipyridine forms a number of clathrates with diphenols; for the quinol clathrate, see: Oswald *et al.* (2005 ▶) and for the 2,2′-biphenol clathrate, see: Lavender *et al.* (1999 ▶). For the crystal structure of 2,4-dihydroxy­benzaldehyde 4-ethyl­thio­semicarbazone, see: Tan *et al.* (2008 ▶).
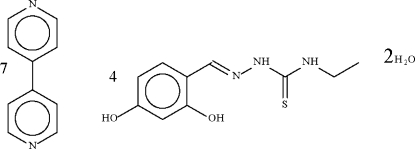


## Experimental

### 

#### Crystal data

7C_10_H_8_N_2_·4C_10_H_13_N_3_O_2_S·2H_2_O
*M* *_r_* = 2086.50
Triclinic, 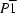

*a* = 11.7683 (3) Å
*b* = 15.2531 (4) Å
*c* = 16.0612 (4) Å
α = 74.639 (2)°
β = 86.561 (2)°
γ = 70.252 (1)°
*V* = 2615.2 (1) Å^3^
*Z* = 1
Mo *K*α radiation
μ = 0.16 mm^−1^
*T* = 153 K
0.20 × 0.20 × 0.05 mm

#### Data collection

Bruker SMART APEX diffractometer
Absorption correction: multi-scan (*SADABS*; Sheldrick, 1996 ▶) *T*_min_ = 0.968, *T*_max_ = 0.992
18419 measured reflections
10042 independent reflections
6419 reflections with *I* > 2σ(*I*)
*R*_int_ = 0.034

#### Refinement

*R*[*F* ^2^ > 2σ(*F* ^2^)] = 0.061
*wR*(*F* ^2^) = 0.179
*S* = 1.02
10042 reflections
686 parameters
170 restraints
H atoms treated by a mixture of independent and constrained refinement
Δρ_max_ = 0.66 e Å^−3^
Δρ_min_ = −0.56 e Å^−3^

### 

Data collection: *APEX2* (Bruker, 2008 ▶); cell refinement: *SAINT* (Bruker, 2008 ▶); data reduction: *SAINT*; program(s) used to solve structure: *SHELXS97* (Sheldrick, 2008 ▶); program(s) used to refine structure: *SHELXL97* (Sheldrick, 2008 ▶); molecular graphics: *X-SEED* (Barbour, 2001 ▶); software used to prepare material for publication: *publCIF* (Westrip, 2009 ▶).

## Supplementary Material

Crystal structure: contains datablocks global, I. DOI: 10.1107/S1600536809030852/ci2864sup1.cif

Structure factors: contains datablocks I. DOI: 10.1107/S1600536809030852/ci2864Isup2.hkl

Additional supplementary materials:  crystallographic information; 3D view; checkCIF report

## Figures and Tables

**Table 1:** Hydrogen-bond geometry (Å, °)

*D*—H⋯*A*	*D*—H	H⋯*A*	*D*⋯*A*	*D*—H⋯*A*
O1—H1o⋯N8^i^	0.84 (4)	1.92 (1)	2.755 (5)	171 (5)
O1—H1o⋯N8′^i^	0.84 (4)	1.97 (1)	2.798 (5)	171 (5)
O2—H2o⋯N7	0.84 (4)	1.93 (1)	2.768 (5)	172 (4)
O2—H2o⋯N7′	0.84 (4)	1.93 (1)	2.766 (5)	178 (4)
O3—H3o⋯N10^ii^	0.84 (4)	1.91 (1)	2.751 (5)	178 (5)
O3—H3o⋯N10′^ii^	0.84 (4)	1.90 (1)	2.736 (5)	172 (5)
O4—H4o⋯N11	0.84 (4)	1.90 (1)	2.735 (4)	174 (4)
O1*w*—H1*w*1⋯O4	0.84 (3)	2.04 (2)	2.843 (4)	160 (5)
O1*w*—H1*w*2⋯N9	0.84 (3)	1.94 (1)	2.782 (5)	175 (7)
O1*w*—H1*w*2⋯N9′	0.84 (3)	1.91 (3)	2.722 (5)	160 (7)
N2—H2n⋯O1*w*	0.88 (3)	1.88 (1)	2.757 (4)	175 (4)

Symmetry codes: (i) 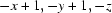; (ii) 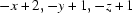.

**Fig. 1.**
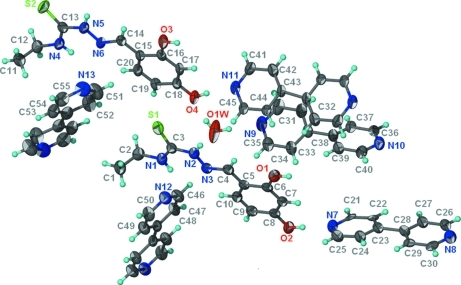
Displacement ellipsoid plot (Barbour, 2001) of 7C10H8N2.4C10H13N3O2S.2H2O at the 70% probability level; H atoms are drawn as spheres of arbitrary radius. Unlabeled atoms in N11-, N12- and N13-pyridine rings are related to other labeled atoms by the symmetry operation (2 - x, 1 - y, 1 - z), (-x - 1, 2 - y, 1 - z) and (-x, 2 - y, 2 - z), respectively. Only one component of the disordered molecules are shown.

## supplementary crystallographic information

### Experimental

2,4-Dihydroxybenzaldehyde ethylthiosemicarbazone (0.48 g, 2 mmol) and 4,4'-bipyridine (0.15 g, 1 mmol) were dissolved in ethanol (20 ml) and the solution was set aside for a week. The solid material that separated from the solution consisted of small, transparent crystals embedded in some yellow, opaque clumps.

### Refinement

Carbon-bound H-atoms were placed in calculated positions (C—H = 0.95–0.98 Å) and were included in the refinement in the riding-model approximation, with *U*(H) set to 1.2 to 1.5*U_eq_*(C). The amino and water H-atoms were located in a difference Fourier map, and were refined with distance restraints (O—H = 0.84 (1) and N—H = 0.88 (1) Å); their *U*_iso_ parameters were freely refined.

The two 4,4'-bipyridine molecules on general positions and one of the three on special positions are disordered over two positions. A consideration of the short C···C contacts necessitated a 1:1 type of disorder. For these, the pyridyl rings were refined as rigid hexagons of 1.39 Å sides as attempts to restrain C—C and C—N distances to appropriate values require an excessive number of restraints. The U^ij^ parameters of the primed atoms were set to those of the unprimed ones. Additionally, the anisotropic displacement parameters of the carbon atoms were tightly restrained so that they were almost nearly isotropic. The C21/C21' atoms have somewhat elongated thermal ellipsoids, which is probably a consequence of the disorder. More than two orientation of the disordered molecules are possible but splitting the molecules further did not yield more meaningful results.

### Crystal data

**Table:**

7(C_10_H_8_N_2_)·4(C_10_H_13_N_3_O_2_S)·2(H_2_O)	*Z* = 1
*M**_r_* = 2086.50	*F*(000) = 1098
Triclinic, *P*1	*D*_x_ = 1.325 Mg m^−^^3^
Hall symbol: -P 1	Mo *K*α radiation, λ = 0.71073 Å
*a* = 11.7683 (3) Å	Cell parameters from 3358 reflections
*b* = 15.2531 (4) Å	θ = 2.6–26.0°
*c* = 16.0612 (4) Å	µ = 0.16 mm^−^^1^
α = 74.639 (2)°	*T* = 153 K
β = 86.561 (2)°	Plate, yellow
γ = 70.252 (1)°	0.20 × 0.20 × 0.05 mm
*V* = 2615.2 (1) Å^3^	

### Data collection

**Table:**

Bruker SMART APEX diffractometer	10042 independent reflections
Radiation source: fine-focus sealed tube	6419 reflections with *I* > 2σ(*I*)
graphite	*R*_int_ = 0.034
ω scans	θ_max_ = 26.0°, θ_min_ = 1.3°
Absorption correction: multi-scan (*SADABS*; Sheldrick, 1996)	*h* = −14→15
*T*_min_ = 0.968, *T*_max_ = 0.992	*k* = −19→19
18419 measured reflections	*l* = −20→20

### Refinement

**Table:**

Refinement on *F*^2^	Primary atom site location: structure-invariant direct methods
Least-squares matrix: full	Secondary atom site location: difference Fourier map
*R*[*F*^2^ > 2σ(*F*^2^)] = 0.061	Hydrogen site location: inferred from neighbouring sites
*wR*(*F*^2^) = 0.179	H atoms treated by a mixture of independent and constrained refinement
*S* = 1.02	*w* = 1/[σ^2^(*F*_o_^2^) + (0.0783*P*)^2^ + 2.0276*P*] where *P* = (*F*_o_^2^ + 2*F*_c_^2^)/3
10042 reflections	(Δ/σ)_max_ = 0.001
686 parameters	Δρ_max_ = 0.66 e Å^−^^3^
170 restraints	Δρ_min_ = −0.56 e Å^−^^3^

### Fractional atomic coordinates and isotropic or equivalent isotropic displacement parameters (Å^2^)

**Table:**

	*x*	*y*	*z*	*U*_iso_*/*U*_eq_	Occ. (<1)
S1	0.25549 (8)	0.84394 (7)	0.73613 (5)	0.0403 (2)	
S2	0.65813 (8)	0.93784 (8)	1.26910 (7)	0.0511 (3)	
O1	0.3505 (2)	0.66350 (18)	0.35305 (17)	0.0458 (6)	
H1O	0.388 (4)	0.645 (3)	0.311 (2)	0.081 (15)*	
O2	−0.0039 (2)	0.74630 (19)	0.17564 (17)	0.0470 (6)	
H2O	0.045 (3)	0.721 (3)	0.141 (2)	0.058 (12)*	
O3	0.9220 (2)	0.67293 (17)	0.94434 (15)	0.0396 (6)	
H3O	0.966 (4)	0.644 (3)	0.910 (3)	0.100 (18)*	
O4	0.6292 (2)	0.70169 (17)	0.73457 (14)	0.0386 (5)	
H4O	0.693 (2)	0.679 (3)	0.710 (2)	0.066 (13)*	
O1w	0.4655 (3)	0.7058 (3)	0.6093 (2)	0.0781 (10)	
H1w1	0.500 (4)	0.716 (3)	0.648 (2)	0.078 (16)*	
H1w2	0.509 (5)	0.696 (5)	0.567 (3)	0.15 (3)*	
N1	0.0466 (2)	0.8843 (2)	0.65301 (18)	0.0353 (6)	
H1N	0.008 (3)	0.877 (3)	0.6112 (16)	0.049 (11)*	
N2	0.2186 (2)	0.7963 (2)	0.59750 (18)	0.0352 (6)	
H2N	0.2980 (10)	0.771 (2)	0.602 (2)	0.049 (11)*	
N3	0.1528 (2)	0.79739 (19)	0.52915 (17)	0.0349 (6)	
N4	0.4992 (2)	0.91841 (19)	1.17051 (18)	0.0343 (6)	
H4N	0.484 (3)	0.904 (3)	1.1236 (14)	0.049 (11)*	
N5	0.6981 (2)	0.8472 (2)	1.14649 (18)	0.0381 (7)	
H5N	0.7738 (13)	0.839 (2)	1.158 (2)	0.042 (10)*	
N6	0.6625 (2)	0.82602 (19)	1.07669 (16)	0.0328 (6)	
C1	−0.0538 (3)	0.8798 (3)	0.7937 (2)	0.0504 (10)	
H1A	−0.0972	0.9212	0.8308	0.076*	
H1B	0.0196	0.8315	0.8244	0.076*	
H1C	−0.1057	0.8472	0.7791	0.076*	
C2	−0.0201 (3)	0.9405 (2)	0.7120 (2)	0.0417 (8)	
H2A	−0.0947	0.9895	0.6818	0.050*	
H2B	0.0297	0.9751	0.7275	0.050*	
C3	0.1660 (3)	0.8421 (2)	0.6589 (2)	0.0313 (7)	
C4	0.2164 (3)	0.7593 (2)	0.4716 (2)	0.0343 (7)	
H4	0.3018	0.7335	0.4791	0.041*	
C5	0.1601 (3)	0.7548 (2)	0.3954 (2)	0.0335 (7)	
C6	0.2295 (3)	0.7075 (2)	0.3359 (2)	0.0362 (8)	
C7	0.1760 (3)	0.7045 (2)	0.2625 (2)	0.0372 (8)	
H7	0.2243	0.6724	0.2228	0.045*	
C8	0.0531 (3)	0.7478 (2)	0.2469 (2)	0.0369 (8)	
C9	−0.0184 (3)	0.7960 (2)	0.3044 (2)	0.0382 (8)	
H9	−0.1030	0.8264	0.2938	0.046*	
C10	0.0360 (3)	0.7987 (2)	0.3766 (2)	0.0371 (8)	
H10	−0.0128	0.8318	0.4155	0.045*	
C11	0.3731 (3)	0.9035 (3)	1.2993 (2)	0.0487 (9)	
H11A	0.3046	0.9408	1.3274	0.073*	
H11B	0.4448	0.8770	1.3379	0.073*	
H11C	0.3542	0.8507	1.2862	0.073*	
C12	0.3977 (3)	0.9690 (2)	1.2159 (2)	0.0384 (8)	
H12A	0.3245	0.9965	1.1775	0.046*	
H12B	0.4147	1.0232	1.2294	0.046*	
C13	0.6134 (3)	0.9001 (2)	1.1925 (2)	0.0345 (7)	
C14	0.7464 (3)	0.7858 (2)	1.0308 (2)	0.0318 (7)	
H14	0.8286	0.7707	1.0467	0.038*	
C15	0.7159 (3)	0.7632 (2)	0.95436 (19)	0.0287 (7)	
C16	0.8071 (3)	0.7075 (2)	0.91068 (19)	0.0291 (7)	
C17	0.7786 (3)	0.6880 (2)	0.83663 (18)	0.0302 (7)	
H17	0.8408	0.6517	0.8065	0.036*	
C18	0.6599 (3)	0.7210 (2)	0.80616 (18)	0.0304 (7)	
C19	0.5682 (3)	0.7757 (2)	0.8492 (2)	0.0338 (7)	
H19	0.4867	0.7987	0.8284	0.041*	
C20	0.5975 (3)	0.7958 (2)	0.9220 (2)	0.0322 (7)	
H20	0.5349	0.8331	0.9512	0.039*	
N7	0.1690 (5)	0.6506 (5)	0.0762 (5)	0.0436 (9)	0.50
C21	0.1857 (6)	0.7027 (3)	−0.0060 (5)	0.0282 (14)	0.50
H21	0.1437	0.7702	−0.0244	0.034*	0.50
C22	0.2638 (7)	0.6562 (5)	−0.0613 (4)	0.0268 (14)	0.50
H22	0.2752	0.6919	−0.1175	0.032*	0.50
C23	0.3253 (5)	0.5576 (5)	−0.0344 (3)	0.0342 (9)	0.50
C24	0.3086 (4)	0.5054 (4)	0.0478 (3)	0.0374 (15)	0.50
H24	0.3506	0.4380	0.0662	0.045*	0.50
C25	0.2304 (5)	0.5519 (4)	0.1031 (3)	0.0419 (15)	0.50
H25	0.2190	0.5163	0.1592	0.050*	0.50
N8	0.5283 (5)	0.4168 (4)	−0.2247 (4)	0.0456 (8)	0.50
C26	0.4775 (4)	0.5168 (4)	−0.2494 (3)	0.0412 (15)	0.50
H26	0.4897	0.5517	−0.3057	0.049*	0.50
C27	0.4088 (4)	0.5656 (3)	−0.1919 (3)	0.0361 (13)	0.50
H27	0.3741	0.6340	−0.2088	0.043*	0.50
C28	0.3910 (5)	0.5145 (5)	−0.1095 (3)	0.0316 (9)	0.50
C29	0.4417 (6)	0.4146 (5)	−0.0848 (4)	0.0283 (14)	0.50
H29	0.4295	0.3796	−0.0285	0.034*	0.50
C30	0.5104 (6)	0.3657 (3)	−0.1423 (5)	0.0322 (15)	0.50
H30	0.5451	0.2974	−0.1254	0.039*	0.50
N7'	0.1582 (5)	0.6579 (5)	0.0664 (5)	0.0436 (9)	0.50
C21'	0.2138 (7)	0.7087 (4)	0.0021 (6)	0.0282 (14)	0.50
H21'	0.1989	0.7753	−0.0039	0.034*	0.50
C22'	0.2911 (6)	0.6620 (5)	−0.0534 (4)	0.0268 (14)	0.50
H22'	0.3291	0.6968	−0.0974	0.032*	0.50
C23'	0.3129 (5)	0.5645 (5)	−0.0447 (4)	0.0342 (9)	0.50
C24'	0.2574 (5)	0.5137 (4)	0.0196 (4)	0.0374 (15)	0.50
H24'	0.2723	0.4471	0.0256	0.045*	0.50
C25'	0.1800 (4)	0.5604 (4)	0.0751 (3)	0.0419 (15)	0.50
H25'	0.1420	0.5257	0.1191	0.050*	0.50
N8'	0.5377 (5)	0.4119 (4)	−0.2166 (5)	0.0456 (8)	0.50
C26'	0.5311 (4)	0.5056 (4)	−0.2200 (3)	0.0412 (15)	0.50
H26'	0.5719	0.5382	−0.2634	0.049*	0.50
C27'	0.4648 (4)	0.5514 (3)	−0.1598 (3)	0.0361 (13)	0.50
H27'	0.4603	0.6154	−0.1621	0.043*	0.50
C28'	0.4051 (5)	0.5037 (5)	−0.0962 (3)	0.0316 (9)	0.50
C29'	0.4117 (6)	0.4101 (5)	−0.0928 (4)	0.0283 (14)	0.50
H29'	0.3709	0.3775	−0.0493	0.034*	0.50
C30'	0.4780 (6)	0.3642 (3)	−0.1530 (5)	0.0322 (15)	0.50
H30'	0.4825	0.3002	−0.1507	0.039*	0.50
N9	0.6183 (5)	0.6645 (4)	0.4755 (3)	0.0440 (18)	0.50
C31	0.6864 (5)	0.7070 (3)	0.4150 (4)	0.0492 (17)	0.50
H31	0.6903	0.7682	0.4152	0.059*	0.50
C32	0.7489 (4)	0.6601 (4)	0.3542 (3)	0.0398 (14)	0.50
H32	0.7954	0.6892	0.3129	0.048*	0.50
C33	0.7432 (5)	0.5706 (4)	0.3540 (4)	0.0336 (12)	0.50
C34	0.6751 (6)	0.5281 (3)	0.4145 (5)	0.0395 (14)	0.50
H34	0.6712	0.4669	0.4143	0.047*	0.50
C35	0.6127 (5)	0.5750 (4)	0.4752 (4)	0.0405 (16)	0.50
H35	0.5661	0.5459	0.5166	0.049*	0.50
N10	0.9335 (5)	0.4261 (4)	0.1649 (3)	0.0391 (8)	0.50
C36	0.8856 (5)	0.5262 (4)	0.1435 (3)	0.0283 (14)	0.50
H36	0.8950	0.5626	0.0871	0.034*	0.50
C37	0.8241 (6)	0.5732 (3)	0.2045 (4)	0.0303 (14)	0.50
H37	0.7914	0.6416	0.1898	0.036*	0.50
C38	0.8105 (5)	0.5199 (3)	0.2869 (4)	0.0314 (10)	0.50
C39	0.8583 (4)	0.4198 (3)	0.3084 (2)	0.0367 (12)	0.50
H39	0.8490	0.3834	0.3648	0.044*	0.50
C40	0.9199 (4)	0.3729 (3)	0.2474 (3)	0.0375 (12)	0.50
H40	0.9526	0.3044	0.2621	0.045*	0.50
N9'	0.6010 (5)	0.6323 (4)	0.4845 (3)	0.0440 (18)	0.50
C31'	0.6291 (5)	0.6924 (3)	0.4109 (4)	0.0492 (17)	0.50
H31'	0.6067	0.7596	0.4057	0.059*	0.50
C32'	0.6898 (5)	0.6541 (4)	0.3448 (3)	0.0398 (14)	0.50
H32'	0.7090	0.6952	0.2945	0.048*	0.50
C33'	0.7225 (6)	0.5558 (4)	0.3525 (4)	0.0336 (12)	0.50
C34'	0.6944 (6)	0.4957 (3)	0.4261 (5)	0.0395 (14)	0.50
H34'	0.7167	0.4285	0.4313	0.047*	0.50
C35'	0.6337 (6)	0.5340 (3)	0.4922 (4)	0.0405 (16)	0.50
H35'	0.6145	0.4929	0.5425	0.049*	0.50
N10'	0.9211 (5)	0.4325 (5)	0.1540 (3)	0.0391 (8)	0.50
C36'	0.9193 (5)	0.5226 (4)	0.1589 (3)	0.0283 (14)	0.50
H36'	0.9624	0.5566	0.1187	0.034*	0.50
C37'	0.8547 (6)	0.5630 (3)	0.2227 (4)	0.0303 (14)	0.50
H37'	0.8535	0.6246	0.2260	0.036*	0.50
C38'	0.7917 (5)	0.5134 (4)	0.2815 (4)	0.0314 (10)	0.50
C39'	0.7934 (4)	0.4233 (3)	0.2767 (3)	0.0367 (12)	0.50
H39'	0.7504	0.3894	0.3169	0.044*	0.50
C40'	0.8581 (4)	0.3829 (3)	0.2129 (3)	0.0375 (12)	0.50
H40'	0.8593	0.3213	0.2096	0.045*	0.50
N11	0.8292 (4)	0.6185 (4)	0.6505 (3)	0.0392 (10)	0.50
C41	0.9111 (4)	0.6576 (3)	0.6025 (3)	0.0398 (12)	0.50
H41	0.9174	0.7158	0.6092	0.048*	0.50
C42	0.9836 (4)	0.6115 (3)	0.5446 (3)	0.0398 (12)	0.50
H42	1.0395	0.6382	0.5118	0.048*	0.50
C43	0.9743 (5)	0.5263 (3)	0.5348 (3)	0.0299 (11)	0.50
C44	0.8924 (5)	0.4872 (3)	0.5828 (4)	0.0270 (14)	0.50
H44	0.8861	0.4290	0.5761	0.032*	0.50
C45	0.8199 (5)	0.5334 (4)	0.6407 (3)	0.0289 (14)	0.50
H45	0.7640	0.5067	0.6735	0.035*	0.50
N11'	0.8058 (5)	0.6149 (4)	0.6454 (3)	0.0392 (10)	0.50
C41'	0.8322 (4)	0.6690 (3)	0.5672 (3)	0.0398 (12)	0.50
H41'	0.8013	0.7375	0.5535	0.048*	0.50
C42'	0.9038 (4)	0.6229 (3)	0.5089 (2)	0.0398 (12)	0.50
H42'	0.9218	0.6599	0.4555	0.048*	0.50
C43'	0.9490 (5)	0.5228 (3)	0.5289 (3)	0.0299 (11)	0.50
C44'	0.9227 (5)	0.4687 (3)	0.6071 (4)	0.0270 (14)	0.50
H44'	0.9536	0.4002	0.6207	0.032*	0.50
C45'	0.8511 (5)	0.5147 (4)	0.6653 (3)	0.0289 (14)	0.50
H45'	0.8330	0.4777	0.7188	0.035*	0.50
N12	−0.1892 (2)	0.9088 (2)	0.56896 (19)	0.0413 (7)	
C46	−0.2279 (3)	0.9691 (2)	0.4914 (2)	0.0421 (8)	
H46	−0.1695	0.9873	0.4538	0.050*	
C47	−0.3472 (3)	1.0070 (2)	0.4621 (2)	0.0392 (8)	
H47	−0.3688	1.0504	0.4064	0.047*	
C48	−0.4356 (3)	0.9812 (2)	0.51455 (19)	0.0306 (7)	
C49	−0.3957 (3)	0.9185 (2)	0.5956 (2)	0.0389 (8)	
H49	−0.4518	0.8986	0.6344	0.047*	
C50	−0.2754 (3)	0.8854 (2)	0.6193 (2)	0.0424 (8)	
H50	−0.2515	0.8431	0.6752	0.051*	
N13	0.3191 (3)	0.8864 (2)	1.0389 (2)	0.0523 (8)	
C51	0.2705 (4)	0.9350 (3)	0.9600 (3)	0.0574 (11)	
H51	0.3237	0.9394	0.9135	0.069*	
C52	0.1478 (3)	0.9795 (3)	0.9415 (2)	0.0518 (10)	
H52	0.1192	1.0120	0.8837	0.062*	
C53	0.0666 (3)	0.9762 (2)	1.0080 (2)	0.0371 (8)	
C54	0.1158 (3)	0.9245 (2)	1.0897 (2)	0.0417 (8)	
H54	0.0647	0.9185	1.1374	0.050*	
C55	0.2396 (3)	0.8816 (3)	1.1015 (2)	0.0479 (9)	
H55	0.2704	0.8462	1.1583	0.057*	

### Atomic displacement parameters (Å^2^)

**Table:**

	*U*^11^	*U*^22^	*U*^33^	*U*^12^	*U*^13^	*U*^23^
S1	0.0363 (5)	0.0501 (5)	0.0309 (4)	−0.0126 (4)	0.0032 (4)	−0.0077 (4)
S2	0.0350 (5)	0.0713 (7)	0.0654 (6)	−0.0167 (5)	0.0108 (4)	−0.0514 (6)
O1	0.0297 (13)	0.0553 (15)	0.0630 (17)	−0.0139 (11)	0.0150 (12)	−0.0363 (14)
O2	0.0386 (14)	0.0649 (17)	0.0536 (16)	−0.0230 (13)	0.0166 (12)	−0.0379 (14)
O3	0.0308 (13)	0.0503 (14)	0.0357 (13)	−0.0050 (11)	0.0080 (10)	−0.0203 (11)
O4	0.0421 (14)	0.0454 (14)	0.0293 (12)	−0.0104 (11)	0.0074 (11)	−0.0181 (11)
O1w	0.0499 (17)	0.119 (3)	0.0486 (17)	0.0148 (16)	−0.0045 (14)	−0.0489 (18)
N1	0.0298 (15)	0.0382 (15)	0.0381 (16)	−0.0083 (12)	0.0068 (12)	−0.0155 (13)
N2	0.0273 (15)	0.0398 (16)	0.0415 (16)	−0.0101 (13)	0.0082 (13)	−0.0184 (13)
N3	0.0344 (15)	0.0342 (15)	0.0413 (16)	−0.0155 (12)	0.0108 (13)	−0.0152 (12)
N4	0.0313 (15)	0.0362 (15)	0.0380 (16)	−0.0069 (12)	0.0050 (12)	−0.0202 (13)
N5	0.0276 (15)	0.0537 (18)	0.0433 (16)	−0.0146 (14)	0.0090 (13)	−0.0302 (14)
N6	0.0349 (15)	0.0376 (15)	0.0318 (14)	−0.0140 (12)	0.0082 (12)	−0.0180 (12)
C1	0.049 (2)	0.050 (2)	0.056 (2)	−0.0161 (18)	0.0219 (19)	−0.0252 (19)
C2	0.0370 (19)	0.0362 (18)	0.053 (2)	−0.0072 (15)	0.0079 (16)	−0.0210 (16)
C3	0.0333 (17)	0.0275 (16)	0.0330 (17)	−0.0116 (13)	0.0083 (14)	−0.0077 (13)
C4	0.0281 (16)	0.0324 (17)	0.0469 (19)	−0.0127 (14)	0.0117 (15)	−0.0165 (15)
C5	0.0312 (17)	0.0316 (16)	0.0450 (19)	−0.0164 (14)	0.0145 (15)	−0.0169 (15)
C6	0.0297 (17)	0.0357 (18)	0.054 (2)	−0.0185 (14)	0.0170 (15)	−0.0222 (16)
C7	0.0340 (18)	0.0379 (18)	0.052 (2)	−0.0184 (15)	0.0189 (16)	−0.0266 (16)
C8	0.0322 (18)	0.0412 (19)	0.049 (2)	−0.0210 (15)	0.0143 (15)	−0.0221 (16)
C9	0.0296 (17)	0.0421 (19)	0.050 (2)	−0.0160 (15)	0.0116 (15)	−0.0213 (16)
C10	0.0342 (18)	0.0355 (18)	0.049 (2)	−0.0144 (15)	0.0177 (15)	−0.0229 (16)
C11	0.044 (2)	0.054 (2)	0.054 (2)	−0.0196 (18)	0.0175 (18)	−0.0236 (19)
C12	0.0279 (17)	0.0398 (19)	0.047 (2)	−0.0040 (14)	0.0074 (15)	−0.0218 (16)
C13	0.0311 (18)	0.0344 (17)	0.0412 (18)	−0.0124 (14)	0.0120 (14)	−0.0157 (15)
C14	0.0295 (16)	0.0340 (17)	0.0360 (17)	−0.0129 (14)	0.0096 (14)	−0.0146 (14)
C15	0.0322 (17)	0.0278 (15)	0.0276 (16)	−0.0114 (13)	0.0086 (13)	−0.0096 (13)
C16	0.0280 (16)	0.0273 (15)	0.0298 (16)	−0.0087 (13)	0.0087 (13)	−0.0063 (13)
C17	0.0347 (17)	0.0282 (16)	0.0251 (16)	−0.0073 (13)	0.0108 (13)	−0.0089 (13)
C18	0.0398 (18)	0.0288 (16)	0.0223 (15)	−0.0128 (14)	0.0074 (13)	−0.0059 (13)
C19	0.0321 (17)	0.0368 (18)	0.0304 (17)	−0.0095 (14)	0.0037 (14)	−0.0084 (14)
C20	0.0332 (17)	0.0287 (16)	0.0320 (17)	−0.0060 (13)	0.0102 (14)	−0.0114 (13)
N7	0.0465 (19)	0.051 (2)	0.040 (2)	−0.0195 (16)	0.0131 (16)	−0.0216 (17)
C21	0.029 (3)	0.0387 (19)	0.031 (2)	−0.0195 (18)	−0.007 (2)	−0.0192 (16)
C22	0.029 (3)	0.0328 (18)	0.0305 (19)	−0.0228 (19)	0.000 (2)	−0.0115 (16)
C23	0.0334 (19)	0.0347 (18)	0.037 (2)	−0.0138 (15)	0.0092 (16)	−0.0115 (16)
C24	0.038 (3)	0.036 (2)	0.038 (3)	−0.012 (2)	0.009 (2)	−0.010 (2)
C25	0.039 (3)	0.049 (2)	0.041 (3)	−0.020 (3)	0.008 (2)	−0.011 (2)
N8	0.0305 (17)	0.0496 (19)	0.059 (2)	−0.0095 (14)	0.0123 (15)	−0.0260 (17)
C26	0.035 (3)	0.050 (2)	0.046 (3)	−0.019 (3)	0.014 (2)	−0.020 (2)
C27	0.036 (3)	0.035 (2)	0.039 (3)	−0.013 (2)	0.009 (2)	−0.011 (2)
C28	0.0282 (19)	0.031 (2)	0.034 (2)	−0.0096 (16)	0.0049 (16)	−0.0064 (17)
C29	0.022 (3)	0.0336 (18)	0.0334 (19)	−0.0144 (19)	−0.004 (2)	−0.0079 (16)
C30	0.021 (4)	0.0358 (18)	0.043 (3)	−0.0053 (18)	−0.012 (2)	−0.0167 (17)
N7'	0.0465 (19)	0.051 (2)	0.040 (2)	−0.0195 (16)	0.0131 (16)	−0.0216 (17)
C21'	0.029 (3)	0.0387 (19)	0.031 (2)	−0.0195 (18)	−0.007 (2)	−0.0192 (16)
C22'	0.029 (3)	0.0328 (18)	0.0305 (19)	−0.0228 (19)	0.000 (2)	−0.0115 (16)
C23'	0.0334 (19)	0.0347 (18)	0.037 (2)	−0.0138 (15)	0.0092 (16)	−0.0115 (16)
C24'	0.038 (3)	0.036 (2)	0.038 (3)	−0.012 (2)	0.009 (2)	−0.010 (2)
C25'	0.039 (3)	0.049 (2)	0.041 (3)	−0.020 (3)	0.008 (2)	−0.011 (2)
N8'	0.0305 (17)	0.0496 (19)	0.059 (2)	−0.0095 (14)	0.0123 (15)	−0.0260 (17)
C26'	0.035 (3)	0.050 (2)	0.046 (3)	−0.019 (3)	0.014 (2)	−0.020 (2)
C27'	0.036 (3)	0.035 (2)	0.039 (3)	−0.013 (2)	0.009 (2)	−0.011 (2)
C28'	0.0282 (19)	0.031 (2)	0.034 (2)	−0.0096 (16)	0.0049 (16)	−0.0064 (17)
C29'	0.022 (3)	0.0336 (18)	0.0334 (19)	−0.0144 (19)	−0.004 (2)	−0.0079 (16)
C30'	0.021 (4)	0.0358 (18)	0.043 (3)	−0.0053 (18)	−0.012 (2)	−0.0167 (17)
N9	0.040 (3)	0.050 (5)	0.037 (2)	−0.005 (3)	0.0048 (17)	−0.017 (3)
C31	0.053 (4)	0.046 (3)	0.040 (2)	−0.002 (3)	0.000 (3)	−0.017 (2)
C32	0.044 (4)	0.036 (2)	0.034 (2)	−0.006 (2)	0.005 (3)	−0.0093 (18)
C33	0.031 (2)	0.034 (2)	0.0305 (16)	−0.0044 (16)	0.0036 (16)	−0.0078 (15)
C34	0.038 (3)	0.041 (3)	0.036 (2)	−0.008 (3)	0.009 (2)	−0.012 (3)
C35	0.035 (3)	0.044 (4)	0.037 (3)	−0.006 (3)	0.005 (2)	−0.012 (3)
N10	0.0273 (17)	0.0492 (19)	0.0391 (18)	−0.0054 (14)	0.0060 (14)	−0.0194 (16)
C36	0.014 (3)	0.043 (2)	0.026 (2)	−0.006 (2)	−0.008 (2)	−0.0083 (18)
C37	0.025 (3)	0.0354 (19)	0.027 (3)	−0.0045 (19)	−0.005 (2)	−0.0084 (18)
C38	0.028 (2)	0.0308 (18)	0.0309 (18)	−0.0036 (16)	0.0038 (16)	−0.0097 (15)
C39	0.038 (3)	0.037 (2)	0.035 (2)	−0.012 (2)	0.0079 (19)	−0.0095 (18)
C40	0.037 (3)	0.036 (2)	0.038 (3)	−0.007 (2)	0.002 (2)	−0.015 (2)
N9'	0.040 (3)	0.050 (5)	0.037 (2)	−0.005 (3)	0.0048 (17)	−0.017 (3)
C31'	0.053 (4)	0.046 (3)	0.040 (2)	−0.002 (3)	0.000 (3)	−0.017 (2)
C32'	0.044 (4)	0.036 (2)	0.034 (2)	−0.006 (2)	0.005 (3)	−0.0093 (18)
C33'	0.031 (2)	0.034 (2)	0.0305 (16)	−0.0044 (16)	0.0036 (16)	−0.0078 (15)
C34'	0.038 (3)	0.041 (3)	0.036 (2)	−0.008 (3)	0.009 (2)	−0.012 (3)
C35'	0.035 (3)	0.044 (4)	0.037 (3)	−0.006 (3)	0.005 (2)	−0.012 (3)
N10'	0.0273 (17)	0.0492 (19)	0.0391 (18)	−0.0054 (14)	0.0060 (14)	−0.0194 (16)
C36'	0.014 (3)	0.043 (2)	0.026 (2)	−0.006 (2)	−0.008 (2)	−0.0083 (18)
C37'	0.025 (3)	0.0354 (19)	0.027 (3)	−0.0045 (19)	−0.005 (2)	−0.0084 (18)
C38'	0.028 (2)	0.0308 (18)	0.0309 (18)	−0.0036 (16)	0.0038 (16)	−0.0097 (15)
C39'	0.038 (3)	0.037 (2)	0.035 (2)	−0.012 (2)	0.0079 (19)	−0.0095 (18)
C40'	0.037 (3)	0.036 (2)	0.038 (3)	−0.007 (2)	0.002 (2)	−0.015 (2)
N11	0.039 (2)	0.0432 (18)	0.0345 (16)	−0.0080 (16)	0.0083 (16)	−0.0186 (14)
C41	0.048 (3)	0.038 (2)	0.037 (3)	−0.012 (2)	0.004 (2)	−0.018 (2)
C42	0.042 (3)	0.035 (2)	0.040 (3)	−0.011 (2)	0.015 (2)	−0.011 (2)
C43	0.034 (3)	0.0284 (17)	0.0249 (17)	−0.0060 (17)	0.0060 (18)	−0.0089 (14)
C44	0.026 (3)	0.031 (2)	0.022 (3)	−0.007 (2)	−0.002 (2)	−0.007 (2)
C45	0.024 (3)	0.040 (3)	0.019 (3)	−0.006 (2)	−0.005 (2)	−0.007 (2)
N11'	0.039 (2)	0.0432 (18)	0.0345 (16)	−0.0080 (16)	0.0083 (16)	−0.0186 (14)
C41'	0.048 (3)	0.038 (2)	0.037 (3)	−0.012 (2)	0.004 (2)	−0.018 (2)
C42'	0.042 (3)	0.035 (2)	0.040 (3)	−0.011 (2)	0.015 (2)	−0.011 (2)
C43'	0.034 (3)	0.0284 (17)	0.0249 (17)	−0.0060 (17)	0.0060 (18)	−0.0089 (14)
C44'	0.026 (3)	0.031 (2)	0.022 (3)	−0.007 (2)	−0.002 (2)	−0.007 (2)
C45'	0.024 (3)	0.040 (3)	0.019 (3)	−0.006 (2)	−0.005 (2)	−0.007 (2)
N12	0.0373 (16)	0.0340 (15)	0.0480 (17)	−0.0078 (13)	0.0078 (13)	−0.0100 (14)
C46	0.040 (2)	0.0369 (19)	0.046 (2)	−0.0140 (16)	0.0115 (16)	−0.0058 (16)
C47	0.0399 (19)	0.0386 (19)	0.0350 (18)	−0.0133 (15)	0.0096 (15)	−0.0046 (15)
C48	0.0381 (17)	0.0237 (15)	0.0291 (16)	−0.0082 (13)	0.0102 (14)	−0.0101 (13)
C49	0.040 (2)	0.0306 (17)	0.0397 (19)	−0.0101 (15)	0.0097 (15)	−0.0031 (15)
C50	0.045 (2)	0.0338 (18)	0.0377 (19)	−0.0054 (16)	0.0063 (16)	−0.0023 (15)
N13	0.0445 (18)	0.0436 (18)	0.058 (2)	−0.0058 (15)	−0.0058 (16)	−0.0050 (16)
C51	0.047 (2)	0.049 (2)	0.059 (3)	−0.0063 (19)	0.009 (2)	−0.002 (2)
C52	0.048 (2)	0.048 (2)	0.042 (2)	−0.0030 (18)	−0.0010 (17)	−0.0004 (17)
C53	0.0438 (19)	0.0246 (16)	0.0378 (18)	−0.0048 (14)	−0.0017 (15)	−0.0076 (14)
C54	0.045 (2)	0.0373 (19)	0.0386 (19)	−0.0060 (16)	0.0005 (16)	−0.0135 (16)
C55	0.051 (2)	0.043 (2)	0.042 (2)	−0.0086 (18)	−0.0096 (18)	−0.0064 (17)

### Geometric parameters (Å, °)

**Table:**

S1—C3	1.686 (3)	C26'—C27'	1.39
S2—C13	1.667 (3)	C26'—H26'	0.95
O1—C6	1.363 (4)	C27'—C28'	1.39
O1—H1O	0.84 (4)	C27'—H27'	0.95
O2—C8	1.370 (4)	C28'—C29'	1.39
O2—H2O	0.84 (4)	C29'—C30'	1.39
O3—C16	1.361 (4)	C29'—H29'	0.95
O3—H3O	0.84 (4)	C30'—H30'	0.95
O4—C18	1.357 (4)	N9—C31	1.39
O4—H4O	0.84 (4)	N9—C35	1.39
O1w—H1w1	0.84 (3)	C31—C32	1.39
O1w—H1w2	0.84 (3)	C31—H31	0.95
N1—C3	1.331 (4)	C32—C33	1.39
N1—C2	1.461 (4)	C32—H32	0.95
N1—H1N	0.88 (3)	C33—C34	1.39
N2—C3	1.357 (4)	C33—C38	1.520 (5)
N2—N3	1.374 (4)	C34—C35	1.39
N2—H2N	0.88 (3)	C34—H34	0.95
N3—C4	1.288 (4)	C35—H35	0.95
N4—C13	1.327 (4)	N10—C36	1.39
N4—C12	1.458 (4)	N10—C40	1.39
N4—H4N	0.88 (3)	C36—C37	1.39
N5—C13	1.369 (4)	C36—H36	0.95
N5—N6	1.370 (4)	C37—C38	1.39
N5—H5N	0.88 (3)	C37—H37	0.95
N6—C14	1.280 (4)	C38—C39	1.39
C1—C2	1.510 (5)	C39—C40	1.39
C1—H1A	0.98	C39—H39	0.95
C1—H1B	0.98	C40—H40	0.95
C1—H1C	0.98	N9'—C31'	1.39
C2—H2A	0.99	N9'—C35'	1.39
C2—H2B	0.99	C31'—C32'	1.39
C4—C5	1.454 (5)	C31'—H31'	0.95
C4—H4	0.95	C32'—C33'	1.39
C5—C10	1.398 (5)	C32'—H32'	0.95
C5—C6	1.405 (4)	C33'—C34'	1.39
C6—C7	1.388 (5)	C33'—C38'	1.517 (5)
C7—C8	1.379 (4)	C34'—C35'	1.39
C7—H7	0.95	C34'—H34'	0.95
C8—C9	1.396 (4)	C35'—H35'	0.95
C9—C10	1.375 (5)	N10'—C36'	1.39
C9—H9	0.95	N10'—C40'	1.39
C10—H10	0.95	C36'—C37'	1.39
C11—C12	1.521 (5)	C36'—H36'	0.95
C11—H11A	0.98	C37'—C38'	1.39
C11—H11B	0.98	C37'—H37'	0.95
C11—H11C	0.98	C38'—C39'	1.39
C12—H12A	0.99	C39'—C40'	1.39
C12—H12B	0.99	C39'—H39'	0.95
C14—C15	1.455 (4)	C40'—H40'	0.95
C14—H14	0.95	N11—C41	1.39
C15—C20	1.393 (4)	N11—C45	1.39
C15—C16	1.405 (4)	C41—C42	1.39
C16—C17	1.385 (4)	C41—H41	0.95
C17—C18	1.385 (4)	C42—C43	1.39
C17—H17	0.95	C42—H42	0.95
C18—C19	1.395 (4)	C43—C44	1.39
C19—C20	1.376 (4)	C43—C43^i^	1.525 (8)
C19—H19	0.95	C44—C45	1.39
C20—H20	0.95	C44—H44	0.95
N7—C21	1.39	C45—H45	0.95
N7—C25	1.39	N11'—C41'	1.39
C21—C22	1.39	N11'—C45'	1.39
C21—H21	0.95	C41'—C42'	1.39
C22—C23	1.39	C41'—H41'	0.95
C22—H22	0.95	C42'—C43'	1.39
C23—C24	1.39	C42'—H42'	0.95
C23—C28	1.565 (6)	C43'—C44'	1.39
C24—C25	1.39	C43'—C43'^i^	1.558 (8)
C24—H24	0.95	C44'—C45'	1.39
C25—H25	0.95	C44'—H44'	0.95
N8—C26	1.39	C45'—H45'	0.95
N8—C30	1.39	N12—C46	1.336 (4)
C26—C27	1.39	N12—C50	1.343 (4)
C26—H26	0.95	C46—C47	1.384 (5)
C27—C28	1.39	C46—H46	0.95
C27—H27	0.95	C47—C48	1.394 (4)
C28—C29	1.39	C47—H47	0.95
C29—C30	1.39	C48—C49	1.392 (4)
C29—H29	0.95	C48—C48^ii^	1.484 (6)
C30—H30	0.95	C49—C50	1.373 (5)
N7'—C21'	1.39	C49—H49	0.95
N7'—C25'	1.39	C50—H50	0.95
C21'—C22'	1.39	N13—C51	1.335 (5)
C21'—H21'	0.95	N13—C55	1.337 (5)
C22'—C23'	1.39	C51—C52	1.384 (5)
C22'—H22'	0.95	C51—H51	0.95
C23'—C24'	1.39	C52—C53	1.392 (5)
C23'—C28'	1.530 (6)	C52—H52	0.95
C24'—C25'	1.39	C53—C54	1.383 (5)
C24'—H24'	0.95	C53—C53^iii^	1.494 (7)
C25'—H25'	0.95	C54—C55	1.381 (5)
N8'—C26'	1.39	C54—H54	0.95
N8'—C30'	1.39	C55—H55	0.95
			
C6—O1—H1O	113 (3)	N8'—C26'—H26'	120.0
C8—O2—H2O	113 (3)	C26'—C27'—C28'	120.0
C16—O3—H3O	106 (4)	C26'—C27'—H27'	120.0
C18—O4—H4O	108 (3)	C28'—C27'—H27'	120.0
H1w1—O1w—H1w2	114 (5)	C29'—C28'—C27'	120.0
C3—N1—C2	122.8 (3)	C29'—C28'—C23'	121.6 (5)
C3—N1—H1N	117 (2)	C27'—C28'—C23'	117.1 (5)
C2—N1—H1N	120 (2)	C28'—C29'—C30'	120.0
C3—N2—N3	121.8 (3)	C28'—C29'—H29'	120.0
C3—N2—H2N	115 (2)	C30'—C29'—H29'	120.0
N3—N2—H2N	123 (2)	C29'—C30'—N8'	120.0
C4—N3—N2	114.8 (3)	C29'—C30'—H30'	120.0
C13—N4—C12	122.8 (3)	N8'—C30'—H30'	120.0
C13—N4—H4N	119 (2)	C31—N9—C35	120.0
C12—N4—H4N	118 (2)	C32—C31—N9	120.0
C13—N5—N6	119.8 (3)	C32—C31—H31	120.0
C13—N5—H5N	116 (2)	N9—C31—H31	120.0
N6—N5—H5N	123 (2)	C31—C32—C33	120.0
C14—N6—N5	116.7 (3)	C31—C32—H32	120.0
C2—C1—H1A	109.5	C33—C32—H32	120.0
C2—C1—H1B	109.5	C32—C33—C34	120.0
H1A—C1—H1B	109.5	C32—C33—C38	119.9 (4)
C2—C1—H1C	109.5	C34—C33—C38	120.1 (4)
H1A—C1—H1C	109.5	C33—C34—C35	120.0
H1B—C1—H1C	109.5	C33—C34—H34	120.0
N1—C2—C1	113.3 (3)	C35—C34—H34	120.0
N1—C2—H2A	108.9	C34—C35—N9	120.0
C1—C2—H2A	108.9	C34—C35—H35	120.0
N1—C2—H2B	108.9	N9—C35—H35	120.0
C1—C2—H2B	108.9	C36—N10—C40	120.0
H2A—C2—H2B	107.7	N10—C36—C37	120.0
N1—C3—N2	117.4 (3)	N10—C36—H36	120.0
N1—C3—S1	124.2 (2)	C37—C36—H36	120.0
N2—C3—S1	118.4 (2)	C36—C37—C38	120.0
N3—C4—C5	121.4 (3)	C36—C37—H37	120.0
N3—C4—H4	119.3	C38—C37—H37	120.0
C5—C4—H4	119.3	C39—C38—C37	120.0
C10—C5—C6	116.9 (3)	C39—C38—C33	119.7 (4)
C10—C5—C4	122.2 (3)	C37—C38—C33	120.3 (4)
C6—C5—C4	120.9 (3)	C38—C39—C40	120.0
O1—C6—C7	121.0 (3)	C38—C39—H39	120.0
O1—C6—C5	118.1 (3)	C40—C39—H39	120.0
C7—C6—C5	120.9 (3)	C39—C40—N10	120.0
C8—C7—C6	120.3 (3)	C39—C40—H40	120.0
C8—C7—H7	119.9	N10—C40—H40	120.0
C6—C7—H7	119.9	C31'—N9'—C35'	120.0
O2—C8—C7	122.4 (3)	C32'—C31'—N9'	120.0
O2—C8—C9	117.3 (3)	C32'—C31'—H31'	120.0
C7—C8—C9	120.3 (3)	N9'—C31'—H31'	120.0
C10—C9—C8	118.7 (3)	C31'—C32'—C33'	120.0
C10—C9—H9	120.6	C31'—C32'—H32'	120.0
C8—C9—H9	120.6	C33'—C32'—H32'	120.0
C9—C10—C5	122.9 (3)	C32'—C33'—C34'	120.0
C9—C10—H10	118.6	C32'—C33'—C38'	120.1 (4)
C5—C10—H10	118.6	C34'—C33'—C38'	119.9 (4)
C12—C11—H11A	109.5	C35'—C34'—C33'	120.0
C12—C11—H11B	109.5	C35'—C34'—H34'	120.0
H11A—C11—H11B	109.5	C33'—C34'—H34'	120.0
C12—C11—H11C	109.5	C34'—C35'—N9'	120.0
H11A—C11—H11C	109.5	C34'—C35'—H35'	120.0
H11B—C11—H11C	109.5	N9'—C35'—H35'	120.0
N4—C12—C11	112.5 (3)	C36'—N10'—C40'	120.0
N4—C12—H12A	109.1	N10'—C36'—C37'	120.0
C11—C12—H12A	109.1	N10'—C36'—H36'	120.0
N4—C12—H12B	109.1	C37'—C36'—H36'	120.0
C11—C12—H12B	109.1	C38'—C37'—C36'	120.0
H12A—C12—H12B	107.8	C38'—C37'—H37'	120.0
N4—C13—N5	115.5 (3)	C36'—C37'—H37'	120.0
N4—C13—S2	124.9 (2)	C37'—C38'—C39'	120.0
N5—C13—S2	119.5 (2)	C37'—C38'—C33'	120.6 (4)
N6—C14—C15	120.0 (3)	C39'—C38'—C33'	119.4 (4)
N6—C14—H14	120.0	C40'—C39'—C38'	120.0
C15—C14—H14	120.0	C40'—C39'—H39'	120.0
C20—C15—C16	118.2 (3)	C38'—C39'—H39'	120.0
C20—C15—C14	121.8 (3)	C39'—C40'—N10'	120.0
C16—C15—C14	120.1 (3)	C39'—C40'—H40'	120.0
O3—C16—C17	122.0 (3)	N10'—C40'—H40'	120.0
O3—C16—C15	117.9 (3)	C41—N11—C45	120.0
C17—C16—C15	120.1 (3)	N11—C41—C42	120.0
C16—C17—C18	120.5 (3)	N11—C41—H41	120.0
C16—C17—H17	119.8	C42—C41—H41	120.0
C18—C17—H17	119.8	C41—C42—C43	120.0
O4—C18—C17	121.7 (3)	C41—C42—H42	120.0
O4—C18—C19	118.1 (3)	C43—C42—H42	120.0
C17—C18—C19	120.2 (3)	C44—C43—C42	120.0
C20—C19—C18	119.0 (3)	C44—C43—C43^i^	109.9 (7)
C20—C19—H19	120.5	C42—C43—C43^i^	129.1 (7)
C18—C19—H19	120.5	C45—C44—C43	120.0
C19—C20—C15	122.1 (3)	C45—C44—H44	120.0
C19—C20—H20	118.9	C43—C44—H44	120.0
C15—C20—H20	118.9	C44—C45—N11	120.0
C21—N7—C25	120.0	C44—C45—H45	120.0
N7—C21—C22	120.0	N11—C45—H45	120.0
N7—C21—H21	120.0	C41'—N11'—C45'	120.0
C22—C21—H21	120.0	C42'—C41'—N11'	120.0
C21—C22—C23	120.0	C42'—C41'—H41'	120.0
C21—C22—H22	120.0	N11'—C41'—H41'	120.0
C23—C22—H22	120.0	C41'—C42'—C43'	120.0
C24—C23—C22	120.0	C41'—C42'—H42'	120.0
C24—C23—C28	126.3 (5)	C43'—C42'—H42'	120.0
C22—C23—C28	112.9 (5)	C42'—C43'—C44'	120.0
C25—C24—C23	120.0	C42'—C43'—C43'^i^	116.6 (6)
C25—C24—H24	120.0	C44'—C43'—C43'^i^	122.0 (6)
C23—C24—H24	120.0	C43'—C44'—C45'	120.0
C24—C25—N7	120.0	C43'—C44'—H44'	120.0
C24—C25—H25	120.0	C45'—C44'—H44'	120.0
N7—C25—H25	120.0	C44'—C45'—N11'	120.0
C26—N8—C30	120.0	C44'—C45'—H45'	120.0
C27—C26—N8	120.0	N11'—C45'—H45'	120.0
C27—C26—H26	120.0	C46—N12—C50	115.3 (3)
N8—C26—H26	120.0	N12—C46—C47	124.4 (3)
C26—C27—C28	120.0	N12—C46—H46	117.8
C26—C27—H27	120.0	C47—C46—H46	117.8
C28—C27—H27	120.0	C46—C47—C48	119.7 (3)
C29—C28—C27	120.0	C46—C47—H47	120.1
C29—C28—C23	112.9 (5)	C48—C47—H47	120.1
C27—C28—C23	126.9 (5)	C49—C48—C47	116.0 (3)
C28—C29—C30	120.0	C49—C48—C48^ii^	122.3 (3)
C28—C29—H29	120.0	C47—C48—C48^ii^	121.7 (3)
C30—C29—H29	120.0	C50—C49—C48	120.0 (3)
C29—C30—N8	120.0	C50—C49—H49	120.0
C29—C30—H30	120.0	C48—C49—H49	120.0
N8—C30—H30	120.0	N12—C50—C49	124.5 (3)
C21'—N7'—C25'	120.0	N12—C50—H50	117.7
C22'—C21'—N7'	120.0	C49—C50—H50	117.7
C22'—C21'—H21'	120.0	C51—N13—C55	115.0 (3)
N7'—C21'—H21'	120.0	N13—C51—C52	124.5 (4)
C21'—C22'—C23'	120.0	N13—C51—H51	117.7
C21'—C22'—H22'	120.0	C52—C51—H51	117.7
C23'—C22'—H22'	120.0	C51—C52—C53	119.7 (3)
C24'—C23'—C22'	120.0	C51—C52—H52	120.2
C24'—C23'—C28'	115.3 (5)	C53—C52—H52	120.2
C22'—C23'—C28'	124.5 (5)	C54—C53—C52	116.3 (3)
C23'—C24'—C25'	120.0	C54—C53—C53^iii^	121.7 (4)
C23'—C24'—H24'	120.0	C52—C53—C53^iii^	122.0 (4)
C25'—C24'—H24'	120.0	C55—C54—C53	119.7 (3)
C24'—C25'—N7'	120.0	C55—C54—H54	120.2
C24'—C25'—H25'	120.0	C53—C54—H54	120.2
N7'—C25'—H25'	120.0	N13—C55—C54	124.8 (3)
C26'—N8'—C30'	120.0	N13—C55—H55	117.6
C27'—C26'—N8'	120.0	C54—C55—H55	117.6
C27'—C26'—H26'	120.0		
			
C3—N2—N3—C4	173.4 (3)	C24'—C23'—C28'—C27'	−178.5 (3)
C13—N5—N6—C14	172.5 (3)	C22'—C23'—C28'—C27'	−4.4 (6)
C3—N1—C2—C1	−86.2 (4)	C27'—C28'—C29'—C30'	0.0
C2—N1—C3—N2	−176.4 (3)	C23'—C28'—C29'—C30'	166.7 (6)
C2—N1—C3—S1	2.2 (4)	C28'—C29'—C30'—N8'	0.0
N3—N2—C3—N1	3.3 (4)	C26'—N8'—C30'—C29'	0.0
N3—N2—C3—S1	−175.4 (2)	C35—N9—C31—C32	0.0
N2—N3—C4—C5	180.0 (3)	N9—C31—C32—C33	0.0
N3—C4—C5—C10	5.7 (5)	C31—C32—C33—C34	0.0
N3—C4—C5—C6	−175.8 (3)	C31—C32—C33—C38	179.4 (6)
C10—C5—C6—O1	−179.5 (3)	C32—C33—C34—C35	0.0
C4—C5—C6—O1	1.9 (4)	C38—C33—C34—C35	−179.4 (6)
C10—C5—C6—C7	−0.4 (5)	C33—C34—C35—N9	0.0
C4—C5—C6—C7	−179.0 (3)	C31—N9—C35—C34	0.0
O1—C6—C7—C8	178.8 (3)	C40—N10—C36—C37	0.0
C5—C6—C7—C8	−0.2 (5)	N10—C36—C37—C38	0.0
C6—C7—C8—O2	−179.6 (3)	C36—C37—C38—C39	0.0
C6—C7—C8—C9	0.7 (5)	C36—C37—C38—C33	−179.9 (6)
O2—C8—C9—C10	179.9 (3)	C32—C33—C38—C39	146.9 (4)
C7—C8—C9—C10	−0.4 (5)	C34—C33—C38—C39	−33.7 (6)
C8—C9—C10—C5	−0.3 (5)	C32—C33—C38—C37	−33.2 (6)
C6—C5—C10—C9	0.7 (5)	C34—C33—C38—C37	146.2 (3)
C4—C5—C10—C9	179.3 (3)	C37—C38—C39—C40	0.0
C13—N4—C12—C11	−80.2 (4)	C33—C38—C39—C40	179.9 (6)
C12—N4—C13—N5	176.5 (3)	C38—C39—C40—N10	0.0
C12—N4—C13—S2	−4.5 (5)	C36—N10—C40—C39	0.0
N6—N5—C13—N4	7.0 (4)	C35'—N9'—C31'—C32'	0.0
N6—N5—C13—S2	−172.1 (2)	N9'—C31'—C32'—C33'	0.0
N5—N6—C14—C15	−178.4 (3)	C31'—C32'—C33'—C34'	0.0
N6—C14—C15—C20	9.0 (5)	C31'—C32'—C33'—C38'	−178.4 (6)
N6—C14—C15—C16	−171.3 (3)	C32'—C33'—C34'—C35'	0.0
C20—C15—C16—O3	−177.7 (3)	C38'—C33'—C34'—C35'	178.4 (6)
C14—C15—C16—O3	2.7 (4)	C33'—C34'—C35'—N9'	0.0
C20—C15—C16—C17	1.3 (4)	C31'—N9'—C35'—C34'	0.0
C14—C15—C16—C17	−178.4 (3)	C40'—N10'—C36'—C37'	0.0
O3—C16—C17—C18	177.3 (3)	N10'—C36'—C37'—C38'	0.0
C15—C16—C17—C18	−1.6 (4)	C36'—C37'—C38'—C39'	0.0
C16—C17—C18—O4	−179.0 (3)	C36'—C37'—C38'—C33'	178.9 (6)
C16—C17—C18—C19	1.0 (4)	C32'—C33'—C38'—C37'	19.7 (7)
O4—C18—C19—C20	179.8 (3)	C34'—C33'—C38'—C37'	−158.7 (3)
C17—C18—C19—C20	−0.2 (4)	C32'—C33'—C38'—C39'	−161.4 (4)
C18—C19—C20—C15	−0.1 (5)	C34'—C33'—C38'—C39'	20.3 (6)
C16—C15—C20—C19	−0.4 (4)	C37'—C38'—C39'—C40'	0.0
C14—C15—C20—C19	179.2 (3)	C33'—C38'—C39'—C40'	−178.9 (6)
C25—N7—C21—C22	0.0	C38'—C39'—C40'—N10'	0.0
N7—C21—C22—C23	0.0	C36'—N10'—C40'—C39'	0.0
C21—C22—C23—C24	0.0	C45—N11—C41—C42	0.0
C21—C22—C23—C28	170.0 (5)	N11—C41—C42—C43	0.0
C22—C23—C24—C25	0.0	C41—C42—C43—C44	0.0
C28—C23—C24—C25	−168.5 (6)	C41—C42—C43—C43^i^	167.5 (10)
C23—C24—C25—N7	0.0	C42—C43—C44—C45	0.0
C21—N7—C25—C24	0.0	C43^i^—C43—C44—C45	−169.7 (8)
C30—N8—C26—C27	0.0	C43—C44—C45—N11	0.0
N8—C26—C27—C28	0.0	C41—N11—C45—C44	0.0
C26—C27—C28—C29	0.0	C45'—N11'—C41'—C42'	0.0
C26—C27—C28—C23	174.9 (6)	N11'—C41'—C42'—C43'	0.0
C24—C23—C28—C29	−4.5 (6)	C41'—C42'—C43'—C44'	0.0
C22—C23—C28—C29	−173.7 (3)	C41'—C42'—C43'—C43'^i^	−166.4 (9)
C24—C23—C28—C27	−179.7 (3)	C42'—C43'—C44'—C45'	0.0
C22—C23—C28—C27	11.1 (6)	C43'^i^—C43'—C44'—C45'	165.6 (10)
C27—C28—C29—C30	0.0	C43'—C44'—C45'—N11'	0.0
C23—C28—C29—C30	−175.6 (5)	C41'—N11'—C45'—C44'	0.0
C28—C29—C30—N8	0.0	C50—N12—C46—C47	0.1 (5)
C26—N8—C30—C29	0.0	N12—C46—C47—C48	−0.9 (5)
C25'—N7'—C21'—C22'	0.0	C46—C47—C48—C49	1.0 (5)
N7'—C21'—C22'—C23'	0.0	C46—C47—C48—C48^ii^	−179.2 (4)
C21'—C22'—C23'—C24'	0.0	C47—C48—C49—C50	−0.4 (5)
C21'—C22'—C23'—C28'	−173.9 (6)	C48^ii^—C48—C49—C50	179.8 (4)
C22'—C23'—C24'—C25'	0.0	C46—N12—C50—C49	0.5 (5)
C28'—C23'—C24'—C25'	174.5 (5)	C48—C49—C50—N12	−0.4 (5)
C23'—C24'—C25'—N7'	0.0	C55—N13—C51—C52	0.8 (6)
C21'—N7'—C25'—C24'	0.0	N13—C51—C52—C53	1.1 (7)
C30'—N8'—C26'—C27'	0.0	C51—C52—C53—C54	−2.2 (5)
N8'—C26'—C27'—C28'	0.0	C51—C52—C53—C53^iii^	179.3 (4)
C26'—C27'—C28'—C29'	0.0	C52—C53—C54—C55	1.4 (5)
C26'—C27'—C28'—C23'	−167.3 (5)	C53^iii^—C53—C54—C55	179.9 (4)
C24'—C23'—C28'—C29'	14.4 (6)	C51—N13—C55—C54	−1.7 (6)
C22'—C23'—C28'—C29'	−171.4 (4)	C53—C54—C55—N13	0.5 (6)

Symmetry codes: (i) −*x*+2, −*y*+1, −*z*+1; (ii) −*x*−1, −*y*+2, −*z*+1; (iii) −*x*, −*y*+2, −*z*+2.

### Hydrogen-bond geometry (Å, °)

**Table:**

*D*—H···*A*	*D*—H	H···*A*	*D*···*A*	*D*—H···*A*
O1—H1o···N8^iv^	0.84 (4)	1.92 (1)	2.755 (5)	171 (5)
O1—H1o···N8'^iv^	0.84 (4)	1.97 (1)	2.798 (5)	171 (5)
O2—H2o···N7	0.84 (4)	1.93 (1)	2.768 (5)	172 (4)
O2—H2o···N7'	0.84 (4)	1.93 (1)	2.766 (5)	178 (4)
O3—H3o···N10^i^	0.84 (4)	1.91 (1)	2.751 (5)	178 (5)
O3—H3o···N10'^i^	0.84 (4)	1.90 (1)	2.736 (5)	172 (5)
O4—H4o···N11	0.84 (4)	1.90 (1)	2.735 (4)	174 (4)
O1w—H1w1···O4	0.84 (3)	2.04 (2)	2.843 (4)	160 (5)
O1w—H1w2···N9	0.84 (3)	1.94 (1)	2.782 (5)	175 (7)
O1w—H1w2···N9'	0.84 (3)	1.91 (3)	2.722 (5)	160 (7)
N1—H1n···N12	0.88 (3)	2.31 (3)	3.024 (4)	138 (3)
N2—H2n···O1w	0.88 (3)	1.88 (1)	2.757 (4)	175 (4)
N4—H4n···N13	0.88 (3)	2.56 (2)	3.315 (4)	144 (3)
N5—H5n···O2^v^	0.88 (1)	2.51 (2)	3.325 (4)	155 (3)

Symmetry codes: (iv) −*x*+1, −*y*+1, −*z*; (i) −*x*+2, −*y*+1, −*z*+1; (v) *x*+1, *y*, *z*+1.
